# CTLA-4 and targeted immunotherapy—a key link in the systemic influence of periodontitis: a mini review

**DOI:** 10.3389/fdmed.2025.1700665

**Published:** 2025-11-27

**Authors:** Abirami Thanigaimalai, Deepak Moses Ravindran, S. K. Balaji, B. Bhuvaneswari, C. SriSanjhanaa

**Affiliations:** 1 Department of Periodontology and Oral Implantology, Tagore Dental College and Hospital, Chennai, India; 2 Department of Periodontology and Oral Implantology, Sri Ramachandra University Dental College and Hospital, Chennai, India

**Keywords:** CTLA-4 protein human, immune checkpoint inhibitor, immunotherapy, porphyromonas gingivalis, periodontitis

## Abstract

Cytotoxic T Lymphocyte Associated Protein 4 is a T cell-associated receptor that serves as an immune checkpoint molecule, downregulating immunosurveillance and propagating immune homeostasis. Periodontitis influences the serum levels of CTLA-4, which in turn alters the T cell activation pathways, PD-1 pathways, and CD80 activation, which has a key role in antigen presentation and implicates the B cell-mediated antibody response in periodontitis. In contrast, this elevation in CTLA-4 affects various other systemic immuno-inflammatory disorders, predominantly cancer and the efficacy of its immunotherapy. In the current article, an extensive literature review is conducted to elicit the link between the elevation of CTLA-4 in periodontitis and its possible influence on systemic immune-inflammatory disorders and their related targeted immune therapies. After investigation, CTLA-4 and its molecular therapy were found to have a crucial role in altering cancer pathogenesis, immunotherapy, and the pathogenesis of autoimmune disorders. The current article elaborates on the immuno-inflammatory pathways, molecular links, and plausible mechanisms linking periodontitis-associated CTLA-4 elevation and CTLA-4-based molecular therapy, specifically focusing on cancer immunotherapy.

## 1 Introduction

Periodontal medicine is a branch of periodontology that investigates the role of periodontal immunoinflammation in the pathogenesis of various systemic conditions. A link between periodontitis and cardiovascular disease and diabetes mellitus has been firmly established. In addition, various other systemic conditions have been associated with periodontitis, such as adverse pregnancy outcomes and chronic obstructive pulmonary disease, but no firm cause has yet been found. This branch of periodontology has been extensively researched for the past 100 years and is still working on identifying newer links focusing on “modern age epidemics” such as cardiovascular diseases, type II diabetes mellitus, rheumatoid arthritis, Alzheimer's disease, and cancer (1).

The global burden of cancer is increasing swiftly, with over 20 million new cases and 9.7 million deaths reported worldwide each year. The World Health Organization survey reports that 1 in 5 people develop cancer in their lifetime, and 1 in 9 men and 1 in 12 women die because of cancer. Despite a wide array of etiologies being reported, cancer management has historically been confined to limited and proven strategies such as chemotherapy, surgery, and radiation therapy (2). However, with the evolution of modern medicine, newer and more targeted therapeutic strategies are being invented and tested, proving their efficacy. Immunotherapy is one of the specific therapeutic strategies invented that was initially implicated as an adjunctive targeted therapy and is now being applied as a standalone.

Immunotherapy is designed to target various critical aspects of our immune system that either facilitate immune evasion by cancer cells or suppress anti-tumor killing mechanisms. Broadly, immunotherapy targets the immune cells, most importantly T-cells and immune checkpoint molecules. Immune checkpoint molecules are co-inhibitory molecules that prevent the proliferation of T helper and cytotoxic cells and the action of T regulatory (Treg) cells, i.e., they target the gatekeeper molecules which immunoedit cancer (3).

## 2 T cell basics in immunotherapy

**Figure 1:**
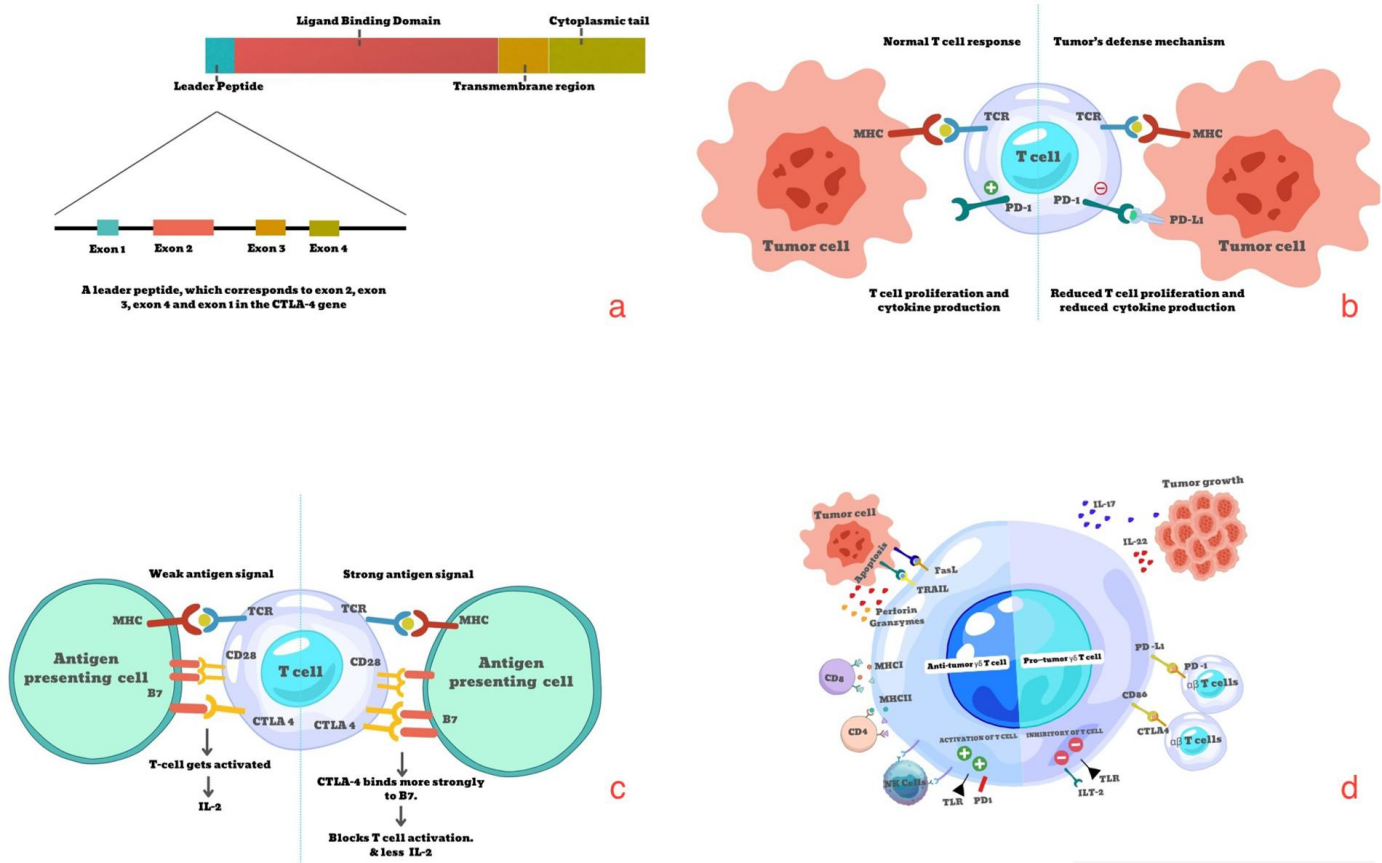
**(a)** Structure of CTLA-4. **(b)** Conventional antigen presentation complex. **(c)** Co-inhibitory signaling of immune checkpoint molecules **(d)** gamma delta T cells. (Original image created using canva software).

T cells are proven to act in a dual role in the progression of cancer, i.e., they can either enhance tumor progression through immunosuppression or consolidate tumor progression through autoimmunity or cancer immunoediting. The former mechanism is established by means of immune checkpoint molecules. As described earlier, they competitively bind to the costimulatory molecules and lead to T cell immune exhaustion (4) (Figures 1b,c).

Apart from the reported T cell subtypes, newer subtypes have been found, i.e., γδ T cells, which have added an important layer of evidence to the understanding of T cell immunosuppression in cancer. These γδ T cells are seen predominantly in the tumor microenvironment in the state of anergy. They can tip toward either side of balance when approaching tumor progression: tumorigenic or anti-tumorigenic (5). (Figure 1d) γδ T cells' ability to detect antigens without the major histocompatibility complex (MHC) marks it as a cell of importance. Conclusively, T cells being harnessed as a part of cancer management, by means of various targets and immune checkpoint molecules, serves a pivotal role in immunotherapy.

## 3 Cytotoxic T lymphocyte-associated protein 4 (CTLA-4) basic structure and function

CTLA-4 is an important co-inhibitory molecule, also known as immune checkpoint molecules, expressed on the surface of T cells for competitive engagement against cluster of differentiation 28 (CD28) to bind with the B7 ligand on antigen-presenting cells. Human CTLA-4 contains a leader peptide, ligand-binding domain, transmembrane region, and cytoplasmic tail. The leader peptide is a variable zone with four exons proposed to bind with the B7 ligand. (Figure 1a) They are located on chromosome 1 q 33–34 region. CTLA-4 by itself or in synergy with other coinhibitory pairs has proven to propagate tumorigenesis through various direct and indirect mechanisms (6).

### 3.1 Direct mechanism

The most common mechanism on which immunotherapy is designed is one in which CTLA-4, being a membrane receptor of cytotoxic T cells, competitively binds to the B7 ligand, as it possesses absolute structural similarity with CD28, which in turn leads to negative signaling and a reduction in the proliferation of T cells and cytokine release. This suppresses the overall immune-susceptible environment. This occurs at an early stage of tumorigenesis (7, 8) (Figure 1).

### 3.2 Elevation of T regulators

CTLA-4 was also detected to be expressed on T reg cells. In the tumor microenvironment, the elevated expression of CTLA-4 on either of the T cell variants could indirectly stimulate Foxp3 expression, which in turn induces T reg differentiation. They, in turn, lead to elevated vascular endothelial growth factor (VEGF) and programmed cell death protein-1 (PD-1). This, along with programmed cell death ligand 1 (PD-L1), causes anergy of T cells in the tumor microenvironment with angiogenesis (9, 10).

### 3.3 Reverse signaling of indoleamine 2,3-dioxygenase (IDO)

CTLA-4 binding with a B7 ligand on the dendritic cell surface can induce a reverse signaling cascade mediated through indoleamine 2,3-dioxygenase (IDO). IDO are proven to upregulate the Treg cells, which can bridge the elevation of B reg cells via CD4+ CD25+ Foxp3T regs, which produce transforming growth factor (TGF-β). This could synergistically elevate IDO as a loop mechanism, which degrades the essential amino acid tryptophan. Tryptophan induces immunotolerance of the T cells, which can lead to tumorigenesis (11–13).

### 3.4 Casitas B-lineage lymphoma protein (Cb1-b)-mediated mechanism

CTLA-4 upregulates the expression of Cb1-b. Studies have reported that elevated Cb-1b expression induced by CTLA-4 can inhibit the Phosphoinositide 3-kinase/Protein Kinase B (PI3K-Akt) signaling pathway, a key regulator of T cell co-stimulation. Apart from its role in pathway blocking, Cb-1b also directly suppresses CD4+ T cell activation by means of an anergy mechanism (14, 15).

These mechanisms in a broader range bring to light that immune checkpoint molecules, especially CTLA-4, play a key role in tumorigenesis. CTLA-4 was the first targeted molecule for drug development through immunotherapy. Fehleisen and Busch were the first scientists who noticed tumor regression in erysipelas infection (16). This further ignited the research work of William Bradley Coley, who is known as the “Father of Immunotherapy”; he first harnessed the immune system to treat bone cancer in 1891 (17, 18). Over the next five decades, an enormous amount of exploration was done in the field of immunology, and 2018 was an important landmark year for immunotherapy, when James Allison and Tasuku Honjo were awarded the Nobel Prize for blocking checkpoint molecules to suppress tumorigenesis (19). To date, various means of immunotherapy have been invented:

1. Adoptive cell therapy
  - Tumor-infiltrating lymphocyte therapy
  - Engineered T cell receptor therapy
  - Chimeric antigen receptor T cell therapy (CAR-T)
2. Immune checkpoint inhibitors targeting
  - CTLA-4
  - PD-1/PD-L1
  - Lymphocyte activation gene (LAG)
  - T-cell immunoglobulin and mucin domain-containing protein 3 (TIM)
  - Circulating Ribonucleic Acid (Cir RNA)
3. Targeted monoclonal antibody therapy
4. Oncolytic virus therapy (T-**vec—Talimogene laherparepvec)**
5. Cancer vaccine
6. Cytokine therapy [Interferon-α and Interleukin-2 (IL-2)]

Among all the mentioned strategies, immune checkpoint blockade has been the most supported mechanism of immunotherapy to date. However, despite its proven efficacy and success rate, there are also drawbacks, primarily immune-related adverse events (irAEs).

## 4 Immune-related adverse events (irAEs) in immune checkpoint blockade therapy

Immune-related adverse events are simply autoimmune-like reactions expressing themselves in various systems and organs. When it comes to CTLA-4-based immunotherapy, over 64% of cases were reported with immune-related adverse events, which are influenced by age, genetics, and other predisposing immunological conditions. These immune reactions could be due to either T cell or B cell reactivity (20–22). The irAEs are exhibited in various forms, i.e., cutaneous, neurotoxic, nephrotoxic, cardiovascular, gastrointestinal, and hepatotoxic, in concordance with the various types of immune checkpoint molecules targeted (Table 1).

**Table 1:** Systemic irAEs pertaining to various types of immune checkpoint blockers.

S. No.	Systemic irAEs	Immunotherapy agent
1	Cutaneous irAEs Maculopapular rash Pruritic Lichenoid dermatitis	60% of anti-CTLA-4 immunotherapy administered patients 45%–59% of anti-PD-1 immunotherapy administered patients 59% −72% in combination immunotherapy
2	Gastro-intestinal tract related irAEs Diarrhea Enteritis	27%–44% of anti-CTLA-4 immunotherapy administered patients
3	Hepatotoxicity Panlobular hepatitis Periventral infiltrating endothelitis Grades of liver damage Aspartate aminotransferase (ASP)/Alanine aminotransferase (ALT) less than three times the upper limit ASP/ALT three to five times the upper limit ASP/ALT five to nine times the upper limit ASP/ALT 10 times the upper limit Fatal liver damage	Seen in 2%–10% of isolated anti-CTLA-4 immunotherapy administered patients 25%–30% in combination immunotherapy
4	Endocrine related irAEs Secondary graves' disease Thyroid disorders(thyrotoxicosis) Hypophysitis Diabetes mellitus	Overall seen in 10% of immune checkpoint blockade
5	Central Nervous System toxicity Neuromuscular disorders Asceptic meningitis Peripheral neuropathy Ocular lesions	Predominantly seen in combination immunotherapy (12.1%) 6.1% of anti-PD-1 immunotherapy administered patients 3.4% of anti-CTLA-4 immunotherapy administered patients
6	Cardiovascular system related irAEs Myocarditis Heart failure Myocardial fibrosis Cardiomyopathy	Overall seen in less than 10% of immune checkpoint blockade
7	Renal toxicity Nephrotoxicity Tubular acidosis	Overall seen in less than 10% of immune checkpoint blockade
8	Respiratory complications Interstitial lung disease	Overall seen in less than 10% of immune checkpoint blockade
9	Oral irAEs Xerostomia Dysgeusia Mucous membrane lesion/vesiculobullous lesions Periodontitis	Commonly associated with dermal, intestinal, and rheumatoid irAEs

Studies have reported periodontitis as an irAE associated with immune checkpoint inhibitor therapy. Chen et al. (2024) proposed that it could occur as a complication of the oral skin axis, which is attributed to the reactivation of M1 macrophages, which could trigger such a response and resultant bone loss (20). However, contrary to this evidence, an alternative hypothesis with extensive literature evidence states that periodontitis could be a confounding entity, altering the course of the outcome of immunotherapy. Various studies have aimed to elaborate on the possibility of periodontitis influencing various immune checkpoint molecules in synergy with its pathophysiology.

## 5 Periodontitis and its role in immune checkpoint blockade

Periodontitis is an immunoinflammatory disorder of microbial origin. The pathophysiology of the progression of periodontitis is initiated by microbial, predominantly bacterial and viral, etiology, leading to recognition of various pattern recognition receptors and a resultant inflammatory cascade. This periodontal immunological cascade has been proven in literature to interfere with or elevate various immune checkpoint molecules and vice versa. This interplay between immune checkpoint molecules and periodontitis is mediated by various aspects of immune molecules, mediators, and pathways of cell activation and cytokine release. These mechanisms plausibly incur the role of periodontitis in altering the efficacy of immunotherapy. The intervening mechanisms could be broadly perio-pathogen-based and periodontal-immunology-based. The next section of this article will elaborate on the key identified pathogen-based and immune-based mechanisms that support the plausibility of periodontitis in altering various aspects of immune checkpoint inhibitor-based immunotherapy by means of immune checkpoint molecules, mainly CTLA-4, PD-1, and PD-L1.

### 5.1 Toll-like receptor-2 (TLR-2)—complement-mediated mechanism

*Porphyromonas gingivalis*, a keystone pathogen of periodontitis, possesses various virulence factors, one of which is gingipains. They are proven to instigate TLR2, which in turn is elicited to induce a crosstalk with the complement system through complement 5a (C5a) on the surface of innate immune cells, namely PMNs, macrophages, basophils, and mast cells. They, in turn, further activate the C3a. This C3a and C5a synergistic link has been shown to interfere with the action of anti-PD-1 monoclonal antibodies and negatively affect immunotherapy (23–25).

### 5.2 *P. gingivalis* lipopolysaccharide (LPS)-mediated mechanisms

The LPS component of the cell wall is one of the key virulence factors of *P. gingivalis*, which is the first molecule that incites an immune reaction in the host. Wang et al. (2024), through a mouse model, evaluated tumor physical and histologic microenvironment characteristics after stimulating T helper cells with LPS. Upon observation, elevated tumor-associated macrophages (TAM), Tregs, TIM, and PD-L1-active CD8+ T cells were reported. This could plausibly dictate the role of LPS in elevated tumor progression and suppression of the efficacy of immunotherapy by elevated PD-1/PD-L1 (26).

### 5.3 *P. gingivalis* outer membrane protein on immunosuppression

Aaoyagi et al. (2000) performed a cell culture study where stimulation of CTLA-4 led to activation of CTLA-4+ CD4+ T cells and suppression of CD28+ CD40+ T cells. Stimulation of peripheral blood mononuclear cells (PBMC) with *P. gingivalis* outer membrane protein (virulence factor) elevated the expression of IL-10 and transforming growth factor–beta. Additionally, upon administration of the anti-CTLA-4 antibody, it showed a suppressed response, leading to overall immunosuppression (27).

### 5.4 Fim A mediated PD-1 expression

Fim A is an active virulence factor of *P. gingivalis*. It has been proven to induce CD11b, T cells, and IL10, which elevate the PD-1 expression in CD4+ T cells. Outer membrane vesicles also increase PD-L1 concomitantly. This synergistic mechanism, as indicated, improves PD-1/PD-L1 binding and concomitant pathway activation of ligation and T cell anergy. This mechanism could directly indicate a periodontally induced systemic elevation of immune checkpoint molecules and, in turn, affect the efficacy of their blockers (28–30).

### 5.5 Alteration of oral gut axis

Various periodontal pathogens are reported to be tissue invasive in nature, whose penetration into the periodontal connective tissue could ultimately lead to their dissemination in the bloodstream and bacteremia. This has been proven to cause significant dysbiosis in gut microflora. *P. gingivalis* specifically has been shown to disturb the gut microbiota. There are various clinical studies reporting a suppressed efficacy of anti-CTLA-4, anti-PD-1, and anti-PD-L1 in patients with altered gut microflora. Dai and colleagues identified nine key species that could possibly modify the response of the system to immune checkpoint inhibitors, namely *Collinsella stercoris, Bacteroidales, Bacteroides mediterraneensis, Prevotella histicola, Enterococcus casseliflavus, Roseburia intestinalis, Ruminococcus bromii, Staphylococcus haemolyticus*, and *Rothia kristinae*. Two out of these nine microorganisms are proven to be synergistically elevated in the presence of elevated *P. gingivalis* levels, i.e., Prevotella histicola and *Ruminococcus bromii*. Additionally, *Ruminococcus bromii* tests positive in the majority of non-responders to anti-PD-1 immunotherapy. This explains the contribution of periodontopathogenic bacteremia in suppressing the efficacy of immune checkpoint blockade therapy (31–33).

### 5.6 Gingipain-mediated negative binding

A culture of CD4+ T cells in *P. gingivalis* gingipain (lys-gingipain)-containing medium caused elevated CTLA-4 T cells and suppressed proliferation of CD45 CD8T cells. It also reduced the binding capacity of anti-CD45 monoclonal antibodies. This action was observed to be reversed when they were re-cultured in gingipain-deprived medium. Furthermore, after 48 h, a significant reduction in CTLA-4+ CD4 T cells was observed, indicating a direct link was elicited in impaired binding of anti-CTLA-4 (34, 35).

### 5.7 Competitive blockade of PD-1 by peptidoglycans

Gregor et al. have proven that human keratinocytes in response to peptidoglycan of *P. gingivalis* upregulated PD-L1 expression in Myeloid Differentiation Primary Response 88 (MyD88) and Receptor Interacting Protein 2 (RIP-2) in a dependent manner. Additionally, an alteration in the MAP2 kinase pathway also jointly elevated PD-1 levels. This mechanism indirectly alters the molecular availability of PD-1 for its binding with anti-PD-1 monoclonal antibody therapy (36, 37).

The previous paragraphs elaborate on the pathogen-mediated mechanisms that could affect the efficacy of immunotherapy; however, the following content summarizes the immune-based mechanisms that could plausibly interfere with the efficacy of immune checkpoint blockade. CTLA-4, PD-1, and PD-L1 were proven to play crucial roles in the progression of periodontitis and cancer. Their role in periodontitis has previously been proven by various mechanisms induced through microorganisms. However, this periodontitis-based elevation of immune checkpoint molecules could interfere with tumorigenesis, and interference in the efficacy of anti-immune checkpoint blockage has been elaborated by various direct T cell-based mechanisms. Apart from T cell-mediated mechanisms, it was proven through two intricate immunological crosstalks.

### 5.8 Inflammasome/IL1/IL18/PD-1 axis

**Figure 2:**
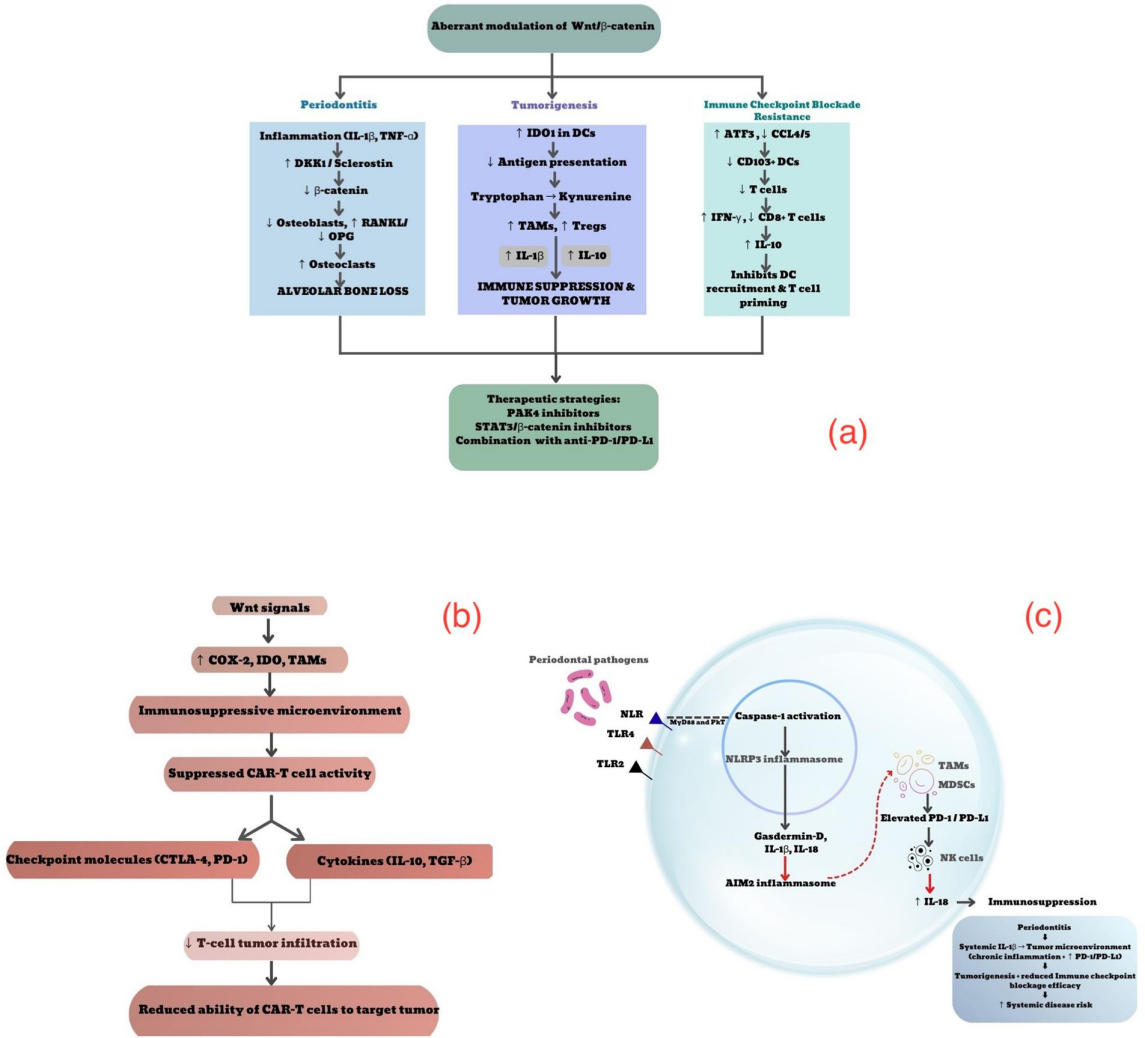
**(a)** Aberrant Wnt/β-catenin pathway induced by periodontitis and its plausible mechanisms in tumorigenesis and resistance to immune checkpoint blockers **(b)** aberrant Wnt/β-catenin pathway on CAR-T cell therapy **(c)** inflammasome-mediated altered efficacy of immune checkpoint blockers induced by periodontitis. (Original image created using canva software).

The first mechanism is mediated through inflammasomes, which are large, complex multiprotein structures formed inside the cells in response to activation of inflammatory responses and cell death. The recognition of periodontal pathogens was primarily through three pattern recognition receptors (PRRs), i.e., TLR-2, TLR-4, and NOD-like receptor (NLR). This, through MyD88 and PkT mechanisms, induces the inflammatory cascade and activation of caspase 1 and, further, NLRP3 inflammasome and secretion of Gasdermin D, IL-1β, and IL-18. These could induce AIM2 inflammasome on the negative balance mechanism, which could elevate the expression of PD-1 and PD-L1 in myeloid-derived suppressor cells (MDSCs) and tumor-associated macrophages (TAMs) (38–40). This, again, on the negative feedback mechanism, could further increase the expression of IL-18 on natural killer (NK) cells, which could lead to immunosuppression. Literature evidence reiterates the role of the IL-1β/PD-1 axis in the accentuation of tumorigenesis and suppression of immune checkpoint blockade (41–43) (Figure 2c).

### 5.9 Aberrant Wnt/β-catenin pathway

Another major inflammatory cascade that could intervene in the inflammatory cascade of tumorigenesis and immune checkpoint blockade is the Wnt/β-catenin pathway. Wnt ligands bind to frizzled (FZD) receptors and low-density lipoprotein receptor protein (LRP5/6) co-receptors and stabilize subsequent nuclear translocation of β-catenin. The first variant of the pathway, i.e., the non-canonical Wnt pathway, is mediated by Wnt5a and Wnt11, FZD, and receptor tyrosine kinases like orphan receptor (ROR1/2) and receptor tyrosine kinase (RTK), and the co-receptor complex, while the canonical pathway trigger mechanisms are independent from β-catenin stabilization. Several proteins can modulate Wnt signaling, such as Dickkopf (DKK) proteins, soluble Frizzled-related proteins (sFRPs), R-spondin proteins, Wnt inhibitory factor-1 (WIF1), and Sclerostin (SOST). Abnormal Wnt/β signaling has been related to several diseases, most significantly cancer and bone pathologies. On one hand, periodontitis-based activation of Wnt/β-catenin could infer its local effect systemically and directly lead to tumorigenesis (44). On the other hand, this abnormality in Wnt/β catenin could activate IDO1 in dendritic cells, deplete their availability for antigen presentation, convert tryptophan to kynurenine, elevate IL-1β, and recruit more TAMs, leading to increased cell survival of T regs, suppression of T cells, and indirect support of tumorigenesis. When focusing on its role in immune checkpoint blockade, an elevated interferon-gamma secretion was seen with concomitant overexpression of beta-catenin, which could deplete CD8+ T cells and cause immune evasion. Additionally, Wnt/β signaling was shown to be inversely efficacious to the immune checkpoint blockers by upregulating activating transcription factor (ATF3), leading to a decrease in CCL4 and CCL5, which are the key chemokines in recruitment of CD103+ DCs and subsequent T cell infiltration. An aberrant Wnt/β-cat can increase the secretion of IL-10, also inhibiting DC recruitment and T cell activation. Inhibition of this metabolic imbalance mediated by Wnt/β-catenin has been shown to improve the efficacy of anti-PD-L1 immunotherapy in a model of mouse melanoma. Inhibition of p21-activated kinase 4 (PAK4), a Wnt-signaling mediator, by genetic deletion or small molecular inhibitors, restored resistance to anti-PD-1 therapy (45–50). Signal transducer and activator of transcription (STAT-3)/Wnt/β catenin signaling inhibitors are now under trial for their use conjugatively with immune checkpoint inhibitors to evade their resistance and improve their efficacy. Conclusively, an activation of aberrant Wnt/β-catenin signaling could elevate tumorigenesis and aid in resistance to immune checkpoint blockers; on the contrary, their blockade could lead to elevated efficacy of immunotherapy (51) (Figures 2a,b).

## 6 Current lacunae and future directions

The described mechanisms indicate periodontitis as a key player whose microbially driven inflammatory cascade could influence both tumorigenesis and immune checkpoint blockade through diverse pathways. Despite the proven mechanisms, we are still unable to infer the direct role of periodontitis on immunotherapy. Research should aim to fill this knowledge gap through definitive evaluation of the confounding nature of periodontitis. Firstly, researchers should evaluate the comprehensive treatment of periodontitis and its further implications in host immune response equilibrium, leading to improved efficacy of immune checkpoint blockers. Secondly, research should focus on key periodontal pathogens to discover their direct role in immune resistance towards immune checkpoint blockade. Thirdly, research should target the key inflammatory mediators of periodontitis, whose neutralization could improve the host response to immunotherapy. Fourthly, the addition or application of anti-inflammatory or probiotic therapy and its efficacy as an adjuvant could also clarify the pathogen-mediated role of periodontitis. Finally, further clarity could be obtained if the role of CTLA-4 was more definitively reiterated in terms of its genetic disturbances, like polymorphisms. Despite the indefinite proof, any systemic immunoinflammatory condition, including periodontitis, could possibly affect the host immune equilibrium. This altered inflammatory state could subdue the host response towards lifesaving cancer immunotherapy. A thorough systemic examination and re-establishment of a neutral inflammatory state should remain key prerequisites for improved efficacy of immunotherapy and overall survival of individuals affected with cancer. The article also recommends investigation into the key role of periodontologists in the prevention of this immune-linked condition and recommends a collaborative role of oncologists and periodontists for a holistic therapeutic outcome.
